# A savanna response to precipitation intensity

**DOI:** 10.1371/journal.pone.0175402

**Published:** 2017-04-07

**Authors:** Ryan S. Berry, Andrew Kulmatiski

**Affiliations:** Department of Wildland Resources and the Ecology Center, Utah State University, Logan, UT, United States of America; Estacion Experimental del Zaidin, SPAIN

## Abstract

As the atmosphere warms, precipitation events are becoming less frequent but more intense. A three-year experiment in Kruger National Park, South Africa, found that fewer, more intense precipitation events encouraged woody plant encroachment. To test whether or not these treatment responses persisted over time, here, we report results from all five years of that experiment. Grass growth, woody plant growth, total fine root number and area and hydrologic tracer uptake by grasses and woody plants were measured in six treated plots (8 m by 8 m) and six control plots. Treatment effects on soil moisture were measured continuously in one treated and one control plot. During the fourth year, increased precipitation intensity treatments continued to decrease water flux in surface soils (0–10 cm), increase water flux in deeper soils (20+ cm), decrease grass growth and increase woody plant growth. Greater root numbers at 20–40 cm and greater woody plant uptake of a hydrological tracer from 45–60 cm suggested that woody plants increased growth by increasing root number and activity (but not root area) in deeper soils. During the fifth year, natural precipitation events were large and intense so treatments had little effect on precipitation intensity or plant available water. Consistent with this effective treatment removal, there was no difference in grass or woody growth rates between control and treated plots, although woody plant biomass remained higher in treated than control plots due to treatment effects in the previous four years. Across the five years of this experiment, we found that 1) small increases in precipitation intensity can result in large increases in deep (20–130 cm) soil water availability, 2) plant growth responses to precipitation intensity are rapid and disappear quickly, and 3) because woody plants accumulate biomass, occasional increases in precipitation intensity can result in long-term increases in woody plant biomass (*i*.*e*., shrub encroachment). While results are likely to be site-specific, they provide experimental evidence of large ecohydrological responses to small changes in precipitation intensity.

## Introduction

Extensive efforts have been made to understand how increases or decreases in total precipitation, as a result of climate change, will affect hydrologic cycles and plant growth [1]. Yet, a more widespread projection of global climate models is that precipitation intensity will increase while total precipitation will remain unchanged [2–4]. More specifically, because the water-holding capacity of air increases by roughly 7% per degree Celsius, it is not unreasonable to expect that precipitation events will become less common but on the order of 25% larger over the next century [3, 5, 6]. Recent observational evidence supports these predictions [7, 8]. Despite the pervasiveness of this projection, experiments testing the effects of precipitation intensity remain limited [9–11]. This is an important gap in understanding because terrestrial productivity, agricultural production, wildfire regimes and biosphere-atmosphere interactions in general are likely to be determined, at least in part, by vegetation responses to changes in precipitation intensity [1, 2, 12].

Semi-arid ecosystems cover over a third of the earth’s surface, produce 30–35% of terrestrial annual net primary productivity (ANPP), support 30% of human populations and most livestock [12, 13] and are particularly sensitive to changes in precipitation intensity [14–16]. Grasslands are common in semi-arid areas and grassland production has been found to be sensitive to changes in precipitation intensity and timing [12, 17]. Shrublands and savannas are also common in semi-arid areas, but less is known about the role of precipitation intensity in semi-arid ecosystems with woody plants. This is an important gap in understanding because over the past 50 years, many semi-arid ecosystems have realized 200 to 1000% increases in woody plant abundance [13, 18, 19]. Fire suppression, increased atmospheric CO_2_ concentrations and decreased grazing have all been suggested encourage woody plant encroachment [18, 20]. A less well recognized and tested explanation for woody encroachment is that more intense precipitation patterns ‘push’ water deeper into the soil providing a competitive advantage to plants with deeper rooting patterns (*i*.*e*., woody plants) [18, 21–23].

Experimental tests of the effects of precipitation intensity are important because ecohydrological models suggest that ecosystem responses may be very sensitive to precipitation intensity [14, 21, 24]. For example, small increases in precipitation intensity may increase plant productivity by decreasing interception and evaporation [25]. Alternatively, large increases in precipitation intensity may decrease plant productivity due to overland flow or deep soil infiltration [24, 26]. Further, factors such as soil type, slope and plant root distributions may also affect plant and water responses to changes in precipitation intensity [27, 28]. Consistent with this understanding of water cycling, aboveground net primary productivity has been found to increase with precipitation intensity in arid grasslands, but decrease with precipitation intensity in mesic grasslands [25]. While a handful of studies do support our understanding of precipitation intensity in North American grasslands, there remains a recognized need for experiments to test the aboveground and belowground effects of increased precipitation intensity in sites with both woody plants and grasses [2, 11, 29, 30]. Further, there is recognized need for multi-year experiments because many of the precipitation intensity experiments that have been performed to date have been limited to single growing seasons [2, 11, 29, 31].

Our objective was to measure both aboveground and belowground responses to increased precipitation intensity in a savanna ecosystem over a five year period. We hypothesized that increased precipitation intensity would ‘push’ water deeper into the soil resulting in increased growth of deeply rooted plants (*i*.*e*., woody plants) and no response or a negative response of shallow-rooted plants (*i*.*e*., grasses). This hypothesis is consistent with previous results, but it was not clear from the first three years of the experiment if positive woody plant growth responses would increase or decrease over time or if grasses would increase growth by increasing rooting depths to better use deeper soil water over time. To test woody plant and grass responses to precipitation intensity, we used a shelter experiment that collected 50% of ambient precipitation and re-deposited that precipitation as relatively large, intense 10 mm events, Kruger National Park, South Africa. Soil moisture, grass growth, tree growth, root image analyses and a hydrologic tracer experiment (used to measure plant water uptake) were used to assess ecosystem responses to increased precipitation intensity treatments. Results from the first three years of the experiment were reported previously [32]. The experiment was maintained for an additional two years and here we report all results from the full five years of the experiment.

## Materials and methods

### Study site

**Fig 1:**
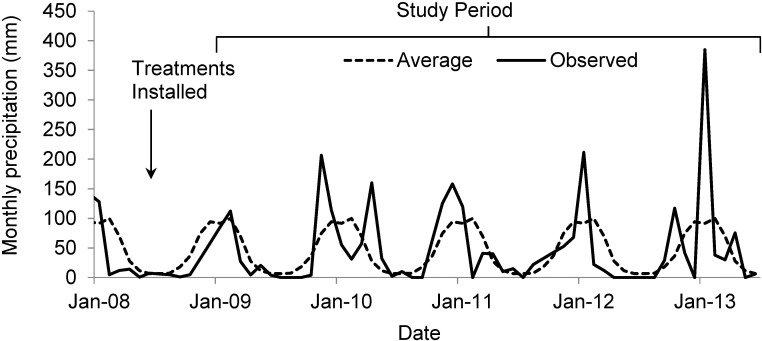
Long-term average (dashed lines) and observed (solid lines) monthly precipitation for Satara Rest Camp, Kruger National Park, South Africa. Treatments were installed March 2008 and ended June 2013. Dates are shown in month-year format.

Research was conducted in the Cape Buffalo enclosure located near the Satara Rest Camp, Kruger National Park, South Africa (24°24’18.30” S, 31°44’52.81”E [33]). Research was approved by South African National Parks and Kruger National Park under project registration number 213896412. Mean annual precipitation is 547 mm, with 356 mm falling December through March (Fig 1; S1 Fig). High radiation values of roughly 18 MJ day^-1^ result in high potential evapotranspiration of roughly 1700 mm yr^-1^ [34, 35]. Climate projections for the region suggest that summer precipitation and precipitation intensity may increase [36, 37]. During the five treatment years in this study (2008–2013), annual precipitation was 459 mm, 654 mm, 473 mm, 388 mm, and 756 mm, respectively. Mean temperatures ranged from highs of 40°C in summer to 8°C in winter. Soils are basalt-derived, dark brown to black pedocutanic clay loams [38]. Common C4 grasses at the study site include *Bothriochloa radicans* (Lehm) A. Camus, *Panicum maximum* (Jacq.), and *Themeda triandra* (Forssk.). Common woody plants include the nitrogen-fixing shrub *Dichrostachys cinerea* subsp. *africana* (Brenan & Brummitt), the tree *Combretum imberbe* (Warwa) and the shrub/tree *Flueggea virosa* (Roxb. Ex Willd.) Voigt. *T*. *triandra*, *D*. *sinerea*, and *F*. *virosa* are common, widespread species in southern Africa. Grass cover at a nearby site was approximately 47% and woody plant cover was approximately 20% [39].

### Experimental design

The experimental design is described elsewhere [32]. Briefly, at the end of the pre-treatment growing season (2007/2008), six 8 m by 8 m by 2.5 m shelters constructed of a steel frame and clear, polycarbonate plastic roofing were erected [33]. Roofing sheets, 13.3 cm wide, covered half the roof and collected 50% of ambient precipitation. The remaining 50% of ambient precipitation fell directly on experimental plots. Collected precipitation was stored in four, 200-L barrels in each shelter. When full, a self-flushing flout mechanism released 10 mm of water over 10–15 minutes through a drip irrigation system that was 60–100 cm above the ground [32]. Treatments were necessarily triggered by natural precipitation events so treatment events only occurred on days with natural precipitation, though treatments were likely to change the timing of large events. Approximately 40% of natural daily precipitation events at the site were historically 10 mm or larger, so treatments represented a relatively small increase in precipitation intensity. Treated plots were paired with untreated plots without roofs [32]. To be clear, throughout the experiment, treatments collected ambient precipitation and deposited that precipitation as relatively large, intense 10 mm events. As a result, total precipitation each year was the same in treated and control plots. Treatment responses were measured during the 2008/2009, 2009/2010, 2010/2011, 2011/2012 and 2012/2013 growing seasons (henceforth, Years ‘1’ through ‘5’, respectively). The site was flat and overland flow was not observed.

#### Soil moisture

Soil moisture was measured at eight depths (5, 10, 20 30, 50, 75, 100 and 130 cm) in one treated and one control plot. Measurements were taken hourly during the growing season and every three hours during the winter. Soil water potential was measured using individually-calibrated heat-dissipation sensors (Campbell Scientific 229 sensors, Logan, UT, USA [32]). Heat dissipation sensors were assumed to measure water potential because salinity was low and osmotic potential was assumed negligible [34]. Soil water was assumed to be plant available (*i*.*e*., PAW) at or above water potentials of -3 MPa. A common midday leaf water potential measured using the chilled mirror technique (WP4T, Decagon Devices, Pullman, USA) at our study site was -2.5 MPa, so -3 MPa was used as a more conservative estimate of PAW [39]. Water potential values were converted to volumetric content using a site-specific soil characteristic curve [38]. PAW calculations were not sensitive to the use of a water potential values close to -3.0 MPa because there is only a small difference in water content (*i*.*e*., 0.008 g g^-1^) between -3.0 MPa and -2.5 MPa [39]. PAW values are used to describe both the pools and fluxes of soil water. Average PAW by depth is reported (S2 Fig), but average PAW values do not distinguish widely variable pools from stagnant pools. For example, a surface soil pool may be wet one day and dry the next, while a deeper soil pool may remain damp both days. In this case, average PAW may be the same at both depths even though more water may have become available at the shallow depths. To provide a better estimate of the soil water that became available at each soil depth, we report the sum of positive PAW increments as PAW flux. For example, if a soil depth contained 0.10 cm cm^-1^ of PAW in one time step and 0.15 cm cm^-1^ of PAW in the next time step, then a positive increment of 0.05 cm cm^-1^ was recorded. This value reveals how much water became available in a soil depth through the year. Gravimetric water contents were converted to volumetric values using soil bulk densities (S1 Table).

#### Aboveground growth (grass)

A disc pasture meter (DPM) measured bulk grass height in treated and control plots [40]. DPM measurements were taken approximately four times annually on 20 or 40 subplots spaced evenly on a grid in each plot (depending on the year). Total grass biomass was harvested and weighed at the end of the second, third and fifth growing seasons. During each biomass harvest, a randomly-selected subsample of about 20, 200 g wet samples from each plot was weighed, dried and re-weighed to develop a dry weight conversion factor which was applied to the remaining wet-collected grass from the plot. Prior to treatments, there was no difference in grass height between control (4.4 ± 0.2 cm) and treated (4.2 ± 0.2 cm) plots [32].

#### Aboveground growth (woody plant)

Sixty woody plants and shrubs (five per plot) were outfitted with small-diameter dendrometer bands (Agricultural Electronics, Tucson, AZ, USA), which were used to measure woody plant circumference increment since last collection date. Across plots, 40 *D*. *cinerea* shrubs, 9 *C*. *imberbe* trees, 5 *Albizia havein* (E. Fourn) trees, 4 *F*. *virosa* shrubs, 1 *Acacia nigrescens* (Oliv. P. J. H. Hurter) tree and 1 *Sclerocarya birrea* (A. Rich Horscht) tree were sampled. All individuals were between 1.8 and 3.1 m tall and stems were between 2.0 and 6.8 cm wide at the start of the experiment. Prior to treatments, stem diameters were similar in control (32.8 ± 3.7 mm) and treated plots (36.6 ± 3.7 mm). Similarly, circumference growth increment was similar in control (4.0 ± 0.3 mm) and treated plots (4.2 ± 0.2 mm). Growth in circumference increment of shrubs (4.0 ± 0.2 mm) and trees (4.3 ± 0.3 mm) were also similar prior to treatments. Hereafter, data from woody plants (trees and shrubs) will be referred to as woody plants for simplicity. Data from woody plants that died during the study were removed because dendrometer installation appeared to damage some woody plants. Dendrometer data was collected approximately four times per year.

#### Plant root responses (rhizotron)

Two-meter-long, clear, cellulose acetate butyrate tubes were installed at a 30° angle in each plot to a vertical depth of roughly 75 cm. Fifty root images (15x magnification) were taken on each of 13 sampling dates from Years 2 through 5 using a BTC-100x video microscope camera (Bartz Technology Co, Carpentaria, CA, USA). The number, diameter, and length of plant roots in each 12.5 by 18 mm minirhizotron ‘window’ in each plot on each sampling date was assessed using RootFly software (Birchfield and Wells, Clemson University, Clemson, SC, USA)[41]. Root area was calculated as the maximum root length times the maximum root width for each root. Root number and area data from images taken over 6.25 cm vertical increments were averaged prior to analyses.

#### Plant root responses (tracer experiment)

To measure vertical patterns of water uptake by grasses and trees in treated and control plots, a tracer experiment was conducted during year Four. Five treated and five control plots were randomly assigned to a target depth (5, 15, 30, 45, and 60 cm) for an early-season (December 2011) and a late-season sampling (April 2012). In each plot, 2,844 pilot holes were drilled to the assigned depth in a 15 cm by 15 cm grid. Two ml of 70% D_2_O tracer (70% deuterium, 30% hydrogen; Cambridge Isotopes, MA, USA) followed by two ml of tap water was injected into each hole using custom needles (16-gauge, thin-wall hypodermic tubing; Vita Needle, Needham, MA, USA), after which each pilot hole was refilled with soil and left for two days [42, 43]. Following the injection and uptake period, non-transpiring grass and woody plant samples were collected in the plots. Grass samples were composited by species so that each sample included plant material from several individuals. Woody plant samples were composited by species and each sample included plant material from several twigs from one to three individuals.

Results from 100 samples from the early-season sampling were published previously [32]. Here we report results from 600 samples from the early- and late-season campaigns. This experiment represented the first time plots were reused in a depth-controlled tracer experiment so it was important to determine if tracer from the first injection persisted in the second injection. To test whether tracer remained in the previously injected depth and to confirm the depth of the new tracer injections, isotope concentrations were measured in soil samples taken one week after injection.

All soil and plant samples from the tracer experiment were immediately sealed with plastic paraffin film in custom-made glass tubes and transported on ice to a freezer within 6 hours. Water was extracted from soil and plant samples by cryogenic distillation within two weeks of sampling, and shipped directly to cold storage [44]. Samples were refrigerated at 4°C until November 2015, where they were analyzed using a cavity ringdown spectrometer (CRDS—Picarro Instruments, Santa Clara, CA, USA). The 100 samples analyzed in 2012 for a previous study were re-analyzed in 2015 for this study and isotope values were highly correlated (R^2^ = 0.97; S3 Fig) indicating that evaporative enrichment had not occurred during sample storage.

### Data analyses and statistics

#### Precipitation and soil moisture

Daily precipitation was collected at the Satara Rest Camp (3 km from the study site) and used to describe precipitation in control plots. Using these data in a ‘tipping bucket’ model to estimate precipitation in treated plots, half the daily precipitation was assumed to land on the plots and half of daily precipitation was assumed to be collected in tanks and deposited once the equivalent of 10 mm of water was collected [45].We report the number of days with precipitation events greater than 2 mm because events less than 2 mm are likely to be lost to interception and evaporation [46]. Both precipitation and PAW data are reported, but not tested statistically because they are taken from one sample.

#### Aboveground growth analyses

Disc pasture meter and woody plant circumference data were analyzed using linear mixed models with a two-way factorial design. For DPM (cm grass height), we used a split plot in time design. For woody plant circumference increment (mm), we used a randomized design with repeated measures. In both cases, fixed effects were treatment (control or increased intensity), date, and the interaction of treatment and date, and plot was a random effect. Time was considered a fixed effect because we were concerned with how our precipitation treatment affected grass, woody plant, over the course of the end of the study. Data were averaged by plot and date, and square root transformed prior to analysis to better meet assumptions of normality.

Grass biomass data (g m^-2^) were analyzed using a linear model with a two-way factorial in a completely randomized design with repeated measures. Fixed effects were treatment (control or increased intensity), date, and the interaction of treatment and date, and plot was a random effect. Time was considered a fixed effect because we were concerned with how our precipitation treatment affected grass, woody plant, over the course of the end of the study. Data were log-transformed prior to analysis to better meet assumptions of normality.

#### Plant root analyses (rhizotron)

Root number and area were analyzed by depth using a linear mixed model. Values were log transformed prior to analysis to meet more adequately assumptions of normality. The fixed effect was treatment and random effects were plots and depth within plots. Data from each year were analyzed separately. Post-hoc pairwise mean comparisons were made to detect differences by treatment and depth using the false discovery rate method to adjust *P* values for Type I error.

#### Plant root analyses (tracer experiment)

Isotope concentrations of plant and soil water samples from the spectrometer were calibrated to standards of known concentration using Picarro Chemcorrect software and reported in delta (δ) notation [42]. To control for natural isotopic enrichment, for example due to evaporation, the deuterium excess relative to ^18^O (D_excess_) was calculated:

$$D_{excess}=D-((8\times^{18}O)+10)$$

where D is the calibrated deuterium value (per mil) for a given sample, and ^18^O is the calibrated ^18^O value for the same sample [39, 47]. D_excess_ in plant samples were used to calculate the proportion of tracer uptake by depth. Proportion and not concentration data were used to control for differences in tracer concentrations among plant species due to traits such as stored water volume [43]. Proportional uptake by depth was calculated for each treatment and functional group (*i*.*e*., 5 cm, control, grass). Proportion of tracer uptake for each sample (***P***_***di***_) was calculated as:

$$P_{di}=\frac{S_{i}-C}{\sum_{a}^{j}{(S}_{d}-C)}$$

where *S*_*i*_ is the *D*_*excess*_ value for each sample, C is the mean *D*_*excess*_ value from control samples, *S*_*d*_ is the mean *D*_*excess*_ value of samples from a soil depth α through *j* (*i*.*e*., *d or* 5 cm). Mean ***P***_***di***_ values by depth are reported. This calculation produces a proportion uptake value for every sample [39, 48]. Finally, each value was converted to a per-cm value by dividing the proportion uptake for a sample by the number of cm that sample represented. For example, samples from the five cm injection depth represented plant uptake from zero cm to 10 cm (*i*.*e*., half of the increment from the five cm injection to the 15 cm injection). This allowed an estimate of the depth at which 50% uptake occurred [39].

To test whether or not tracer was injected to target depths and whether or not tracer from the first campaign was present during the second campaign, we measured tracer concentration in the soil by depth in all plots. To test for differences in tracer concentration among plots, sample depth was standardized:

$$S=d_{n}-d_{p}$$

where *S* is the standardized depth increment, *d*_*n*_ is the depth from which the sample was taken, and *d*_*p*_ is the pulse injection depth (*i*.*e*., a sample from 5–10 cm where injection depth was 5 cm became 0–5 cm). This allowed us to compare uptake proportions by soil depth for each plot. Proportions were calculated as:

$$P=\frac{D_{excess}}{D_{total}}$$

where *D*_*total*_ is the summed *D*_*excess*_ across all samples in a given plot date. Proportions were averaged at each depth across all plots for both campaigns. Proportional tracer uptake by depth was meant to peak at 0 cm after standardization. Differences in tracer concentration among depths were tested using a one-way linear mixed model with a first order autoregressive covariance structure to accommodate spatial autocorrelation among depths. Data were log transformed prior to analysis to better meet assumptions of normality.

Because there was only one plot assigned to each target depth, results from this tracer experiment do not provide inference to water uptake on the landscape. Rather, results provide inference only to the plots in this experiment. Samples within plots, therefore, were used as replicates of each experimental plot. Plant-derived tracer data was grouped by injection campaign (early- or late-season), functional group, and treatment. Data were analyzed using a linear mixed model with fixed effects of treatment type, depth, and the interaction of treatment and depth. All statistical analyses were performed in SAS JMP 9.4 (SAS Institute, Inc., Cary, NC, USA). Differences are considered significant at the α = 0.05 level throughout.

## Results

### Precipitation and soil moisture

**Fig 2:**
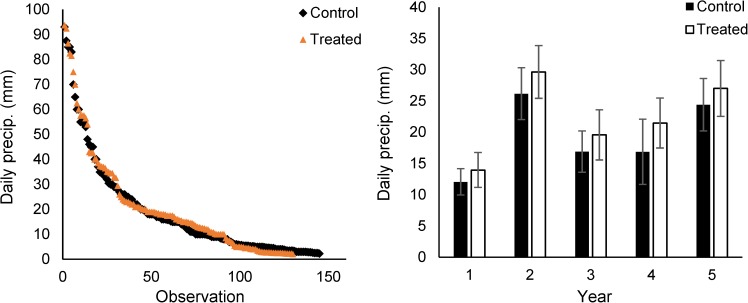
**Treatment effects on (a) the distribution of daily precipitation (days with > 2 mm) in treated and control plots and (b) mean daily precipitation (days with > 2mm) across the five years of the experiment.** Broadly, treatments, decreased the number of days with precipitation by 17% and increased the amount of daily precipitation by 16%.

**Fig 3:**
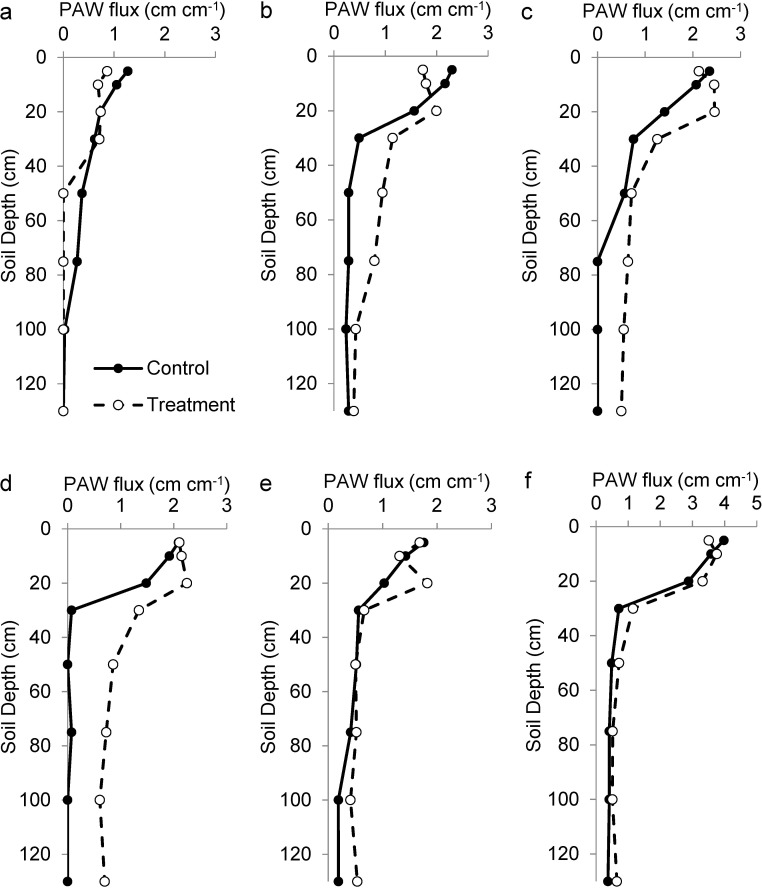
** Annual plant available soil water (PAW) that moved (flux) through the soil in one treated and one control plot for the (a) pretreatment and (c-f) subsequent five treatment years**. Water was assumed plant available when Ψ > -3 MPa. Values represent the sum of positive increments of PAW across each growing season. Note the larger scale during the last season (f). Broadly, treatments, which increased precipitation intensity but not amount, increased deep soil water but did not increase surface (*i*.*e*., 5 cm) soil water.

Across the five years of treatments, there were 145 and 125 days with precipitation (> 2mm) in control and treated plots, respectively (Fig 2A). Average daily precipitation (> 2 mm) was 18.9 and 21.8 mm in control and treated plots, respectively (Fig 2B). Thus, treated plots received 17% fewer days with precipitation, but those days deposited 16% more precipitation than in control plots; total precipitation was the same in control and treated plots. In Years 1–5, mean precipitation on days with precipitation (>2mm) was 16, 13, 16, 27 and 11% larger in treated than control plots (Fig 2B; S1 Fig). Across the experiment, control and treated plots received 74 and 87 days with precipitation > 10 mm, respectively. Consistent with this, PAW flux was 42, 68, 102, 27 and 27% greater in treated than control plots for Years 1–5, respectively (Fig 3B–3F). In contrast, PAW flux was 20% smaller in treated than control plots in the pre-treatment year (Fig 3A). Results for PAW were similar (S2 Fig). Again, ‘PAW flux’ indicated the sum of positive increments of plant available water while ‘PAW’ was the average amount of plant available water. These measures were used to distinguish the amount of water flowing through a soil depth from the amount of water available in a soil depth (i.e., the flux from the pool).

### Aboveground growth

#### Grass

**Fig 4:**
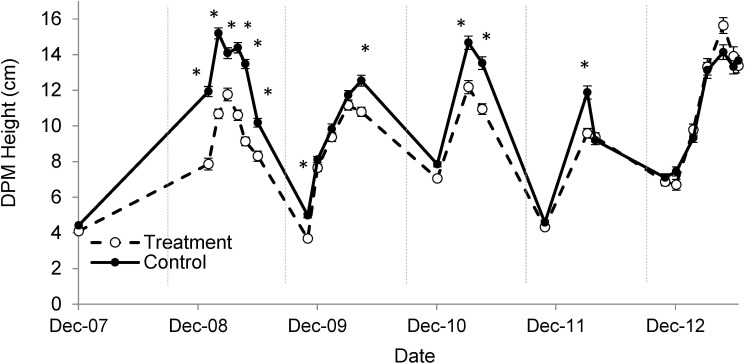
Grass height measured using a disc pasture meter (DPM) in treated plots with increased precipitation intensity (dashed lines, open circles) and control plots with ambient precipitation intensity (solid lines, filled circles). Vertical dotted lines separate growing seasons. Mean ± 1 S.E. shown. Significant differences between treated and control averages are denoted with an asterisk (α = 0.05).

DPM measurements were greater in control than treated plots on several dates in Years 1, 2 and 3 and one date early in Year 4 (Fig 4). Grass biomass was not different in control (185 ± 7 g m^-2^) and treated plots (162 ± 14 g m^-2^; *P* > 0.05).

#### Woody plant

**Fig 5:**
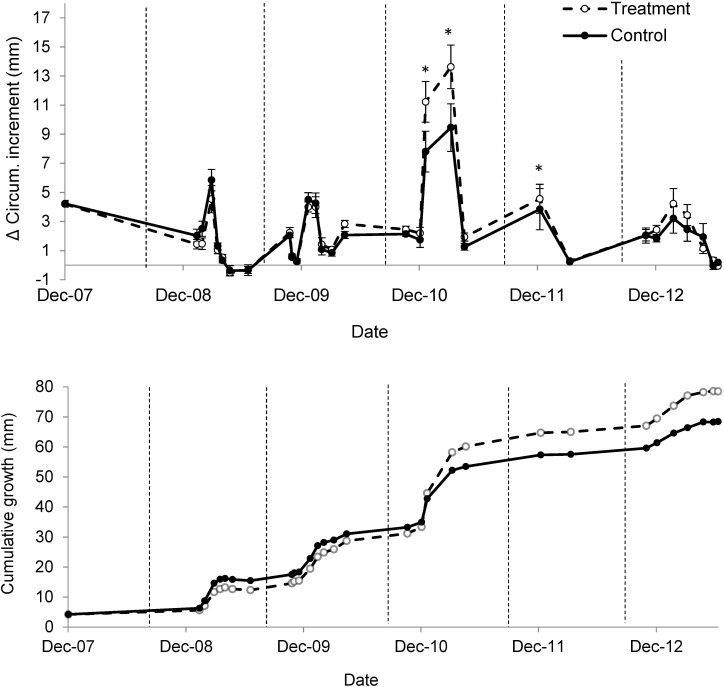
**Circumference increment (a) and cumulative circumference increment (b) of woody plants in treated plots with increased precipitation intensity (dashed lines, open circles), and control plots with ambient precipitation (solid lines, filled circles).** Vertical dotted lines separate growing seasons. Mean ± 1 S.E. shown. Significant differences between treated and control averages are denoted with an asterisk (α = 0.05).

Circumference increment was greater in treated than control plots in Years 3 and early in Year 4 (Fig 5). This resulted in 16% greater cumulative circumference increment in treated than control plots by the end of Year 5 (Fig 5B).

### Root growth

**Fig 6:**
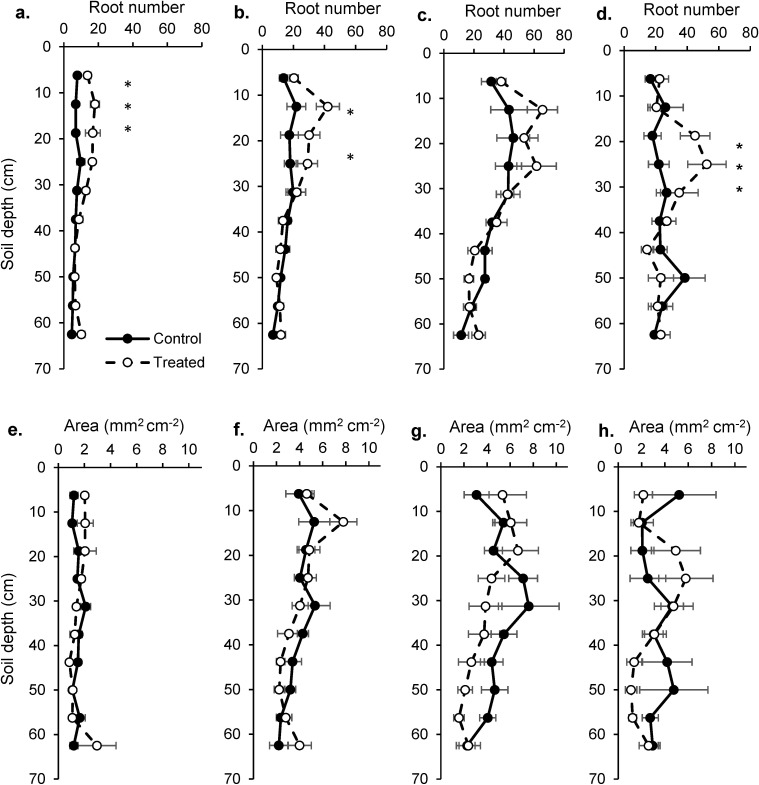
**Number of roots (a-d) and root area (e-g) by depth in treated (dashed lines, open circles) and control plots (solid lines, filled circles) during Year 2 (a, e), 3 (b, f), 4 (c, g) and 5 (d, h) years of the study**. Root number is the sum of the number of roots in five 12.5 by 18 mm windows. Root area is the mean root area (mm^2^) in the same five windows. Mean ± 1 S.E. Asterisks indicate differences between treated and control values at the α = 0.05 level.

During the 13 sampling dates (Years 2 through 5), 27,717 roots were identified. For root number, an interaction between treatment and depth was found in Year 2, 3 and 5 reflecting greater root numbers at 20–40 cm depths in treated than control plots (Fig 6A). No difference was detected in Year 4. In contrast, there was no difference in root area between treated and control plots in any year.

#### D_2_O tracer

**Fig 7:**
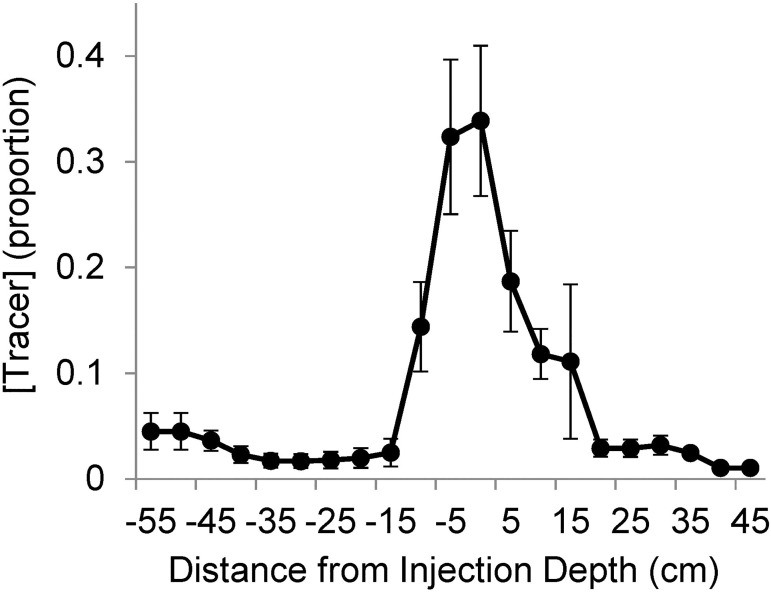
The proportion of tracer concentrations ([tracer]) relative to target injection depth. Positive numbers on the x-axis indicate soil depths beneath the target injection depth, negative numbers indicate soil depths above the target injection depth. Mean ± 1 S.E.

Across treatments, 197 grass samples, 316 woody plant samples, and 87 soil samples were analyzed for isotope concentrations. The large majority of tracer was found within ±5 cm from the target depth (Fig 7).

#### Grass uptake

**Fig 8:**
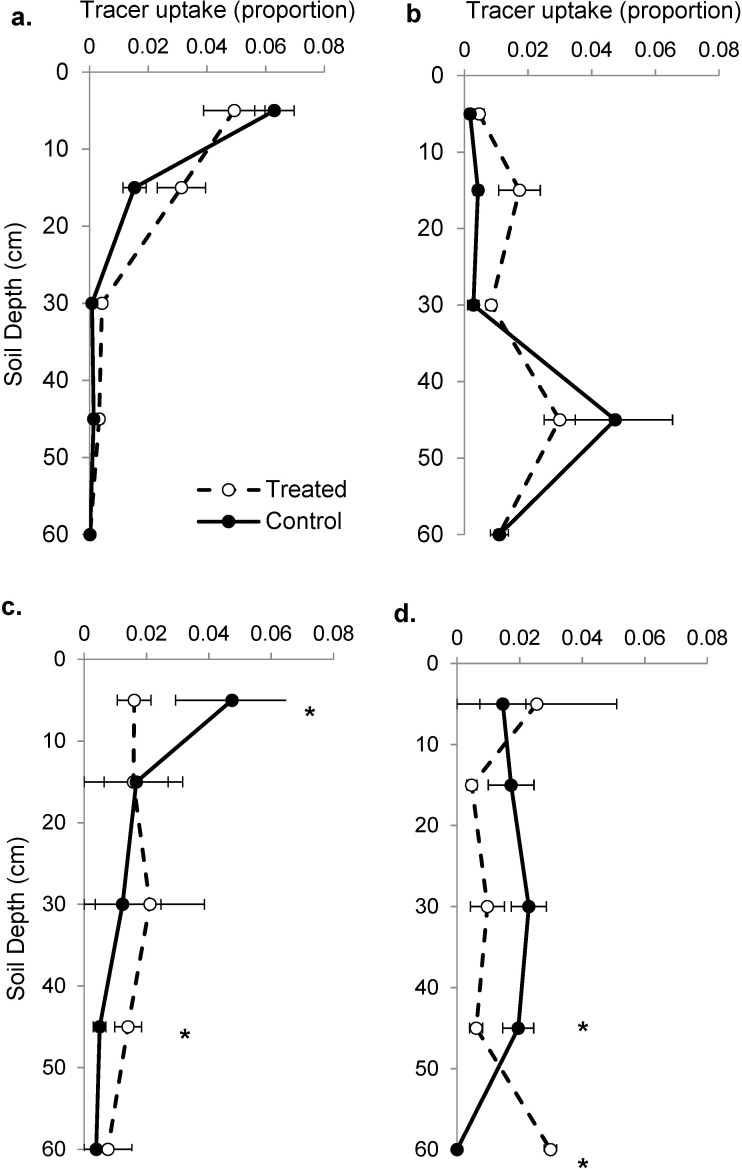
The proportion of deuterium tracer uptake per cm in treated and control plots during the Fourth year of the experiment. Results shown for grasses (a, b) and trees (c, d), in the early-season (a, c) and late-season (b, d). Treated plots indicated with dashed lines, open circles and control plots indicated with solid lines, filled circles. Mean ± 1 S.E. shown. Asterisks indicate significant differences at the α = 0.05 level at the indicated depth.

There was no difference in tracer uptake between treated and control plots at any soil depth either early- or late-season (Fig 8A–8B). In the early season, 50% of grass uptake occurred in the top 8 and 10 cm in control and treated plots, respectively. In the late season, 50% of tracer uptake was deeper and occurred in the top 45 and 41 cm, respectively.

#### Woody plant uptake

Early-season woody plants in treated plots absorbed less tracer from 5 cm and more tracer from 45 cm than woody plants in control plots (Fig 8C). Late-season Woody plants in treated plots absorbed less tracer from 45 cm and more tracer from 60 cm than woody plants in control plots (Fig 8D). Early-season, 50% of woody plant uptake occurred in the top 12 and 29 cm in control and treated plots, respectively. Late-season, 50% of tracer uptake was deeper and occurred in the top 28 and 45 cm in control and treated plots, respectively.

## Discussion

Results from Years 1 through 3, reported previously [32], revealed that small increases in precipitation intensity ‘pushed’ water deeper into the soil profile, decreased grass growth and increased woody plant growth. Here we report results from an additional two years of the same experiment (*i*.*e*., Years 4 and 5). During Year 4, treatments continued to increase precipitation intensity, increase deep plant available water, decrease grass growth and increase woody growth. During Year 5, most natural precipitation events were greater than 10 mm. As a result, experimental treatments that deposited precipitation as 10 mm events had little effect on plant available water. During this last year of the study, when treatments effectively stopped, differences in grass and woody growth between treated and control plots disappeared. However, because woody plants can accumulate biomass, they remained 16% larger in treated than control plots at the end of the experiment. Much of this response was caused by large woody plant growth in Year 3 when treatment effects on soil water were greatest. Broadly, results showed that small increases in precipitation intensity can cause large increases in soil water availability that are likely to benefit woody plants in savannas.

During the five seasons of this study, 39% of daily precipitation values were greater than 10 mm in control plots. Thus, treatments, which deposited 10 mm of water, did not create extreme precipitation events. Rather, treated plots received, on average, 22 mm of precipitation on 125 days, while control plots received 19 mm of precipitation on 145 days (all plots received the same total amount of precipitation; Fig 2). Treatment effects of this magnitude (16% larger daily precipitation) are likely to be realized in the coming decades [2–6]. Despite modest effects on precipitation event size, treatments caused surprisingly large increases in soil water infiltration and availability [21, 24].

Rapid grass responses to treatments provided insight into interactions between grasses and woody plants. The fact that grass growth was smaller in treated plots during the first two years of the experiment, prior to an increase in woody plant growth, suggested that grasses responded directly to soil water availability and not indirectly as a result of competition from woody plants. Similarly, the fact that grass growth did not differ between treated and control plots during the last year of the experiment suggested that the greater woody plant biomass that had accumulated in treated plots had little effect on grass growth. This is consistent with previous research in Kruger National Park that has shown little competitive effect of woody plants on grasses [33]. Recent studies in Kruger have found that grasses obtain roughly 50% of their soil water from the top 15 cm [39, 42, 43], so the decrease in grass growth observed in treated plots was likely caused by a decrease in water flux in surface soils (Fig 3).

In contrast to grasses, woody growth increased in treated plots. This is consistent with recent studies which have found that woody plants rely on water just below that used by grasses (*i*.*e*., 15–30 cm)[39, 42, 43]. Woody growth responses were largely constrained to Year 3, which had the greatest increase in deep soil water availability. The fact that treatment effects on woody growth disappeared quickly at the end of Year 4 suggested a minimal-intensity threshold for woody plant responses rather than a long lag time for woody plant responses to treatment. It should be noted that all of our woody plant samples were from small trees or shrubs so our inference on the response of large trees is limited. Similarly, we do not know the age of the sampled woody plants so we have no inference on the effects of age or size on woody plant responses to treatments. However, there is reason to believe that responses observed in this study will apply to a wide range of woody plants because recent isotope tracer experiments in Kruger Park have shown that even one-year old saplings can realize deeper rooting patterns than large, mature trees [42].

Root analyses helped explain grass and woody responses to increased precipitation intensity. Root image analyses revealed greater root numbers in the 20–40 cm depths in treated than control plots. Several hydrologic tracer studies in Kruger Park suggest that woody plants often demonstrate maximum root activity in these depths [39, 42, 43]. Greater root numbers in treated soils is consistent with the idea that plants were ‘foraging’ for the additional water that became available in treated plots. However, plant available water increased through much of the soil profile, yet root number increased only in the 12.5–25 cm depths. An emphasis by plants on root production in relatively shallow soils is consistent with the minimum rooting depth hypothesis [49]. The fact that root number but not root area increased with treatments suggested that plants relied on changes to the number and location of fine roots and not changes in total root area. It should be noted that grasses dominate root production in this system [50]. As a result, root image data likely primarily reflected grass and not woody responses.

To provide a better understanding of woody root activity, we performed a tracer injection experiment. We are aware of only one study that has combined depth-specific tracer data with root image analyses and that study included 100 tracer samples and two years of root image data [32]. Analyses here used 600 tracer samples and four years of root image data. The tracer experiment was performed in Year 4 when treatments had small effects on plant available water. Consistent with this small treatment effect, root number, root area and grass tracer uptake did not differ between treated and control plots. Tracer data did, however, reveal that woody plants absorbed less shallow and more deep water in treated than control plots in both the early- and late-season samplings. The ability of woody plants to increase deep water uptake presumably allowed them to take advantage of increased infiltration resulting from increased precipitation intensity treatments even in a weak treatment season.

Semi-arid ecosystems are known to be highly sensitive to changes in the timing and amount of precipitation [22]. Results from this experiment showed that even small increases in precipitation intensity can result in large increases in plant available soil water and subsequent plant growth [51]. More specifically, greater precipitation intensity can increase woody plant growth by driving water deeper into the soil where it is less available to grass roots [30]. Our study provides evidence that savannas respond quickly to small increases in mean precipitation intensity, but because woody plants can accumulate the effects of these short-term benefits, that occasionally intense precipitation events caused by climate change are likely to increase shrub encroachment at least until these effects interact with factors such as fire and herbivory [23].

This study was performed in a savanna with fairly level clay soils and roughly 540 mm of summer-dominated precipitation. It is likely that treatment responses will differ in sites with sandy soils, greater precipitation or steeper slopes [33]. Further experiments in a range of conditions will be needed to better parameterize ecohydrological models that can be used to simulate and predict savanna responses to increased precipitation intensity [30, 39]. Similarly, longer-term experiments and different modeling approaches are likely to be needed to understand interactions between climate-driven effects on grass and woody growth and longer-term processes such as fire and herbivory [23].

## Supporting information

S1 Table Soil bulk density (g cm^-3^) and soil porosity (cm^3^ cm^-3^) assumed for soils in the experiment. (DOCX)

S1 Fig Daily precipitation (mm) in control and treated plots. Treatment values are modeled assuming that storage tanks dispensed collected ambient precipitation when full. The dotted horizontal line indicates 10 mm events.
(DOCX)

S2 Fig Mean annual plant available soil water (PAW) by depth in one treated and one control plot for the (a) pretreatment and (c-f) subsequent five treatment years. Water was assumed plant available when Ψ > -3 MPa. Values represent the mean PAW across each growing season (October through March). Broadly, treatments, which increased precipitation intensity but not amount, increased deep soil water but did not increase surface (*i*.*e*., 5 cm) soil water.
(DOCX)

S3 Fig Comparison of isotope samples measured for (a) Deuterium concentration and (b) Deuterium excess above ^18^O values and ambient isotope concentrations. Samples were measured repeatedly 3 years apart.
(DOCX)

S1 File Metadata file. (DOCX)

S2 File Bulk density and porosity data. (XLSX)

S3 File Daily rainfall data. (XLSX)

S4 File Dendrometer data. (XLSX)

S5 File Disc pasture meter data. (XLSX)

S6 File Rhizotron data. (XLSX)

S7 File Isotope tracer data. (XLSX)

## References

[1] Wu Z, Dijkstra P, Koch GW, Pañuelas J, Hungate BA. Responses of terrestrial ecosystems to temperature and precipitation change: a meta-analysis of experimental manipulation. Global Change Biol. 2011;17(2):927–42.

[2] Beier C, Beierkuhnlein C, Wohlgemuth T, Penuelas J, Emmett B, Körner C, et al Precipitation manipulation experiments–challenges and recommendations for the future. Ecol Lett. 2012:n/a-n/a. 10.1111/j.1461-0248.2012.01793.x 22553898

[3] Trenberth KE. Changes in precipitation with climate change. Clim Res. 2011;47(1):123.

[4] Molnar P, Fatichi S, Gaál L, Szolgay J, Burlando P. Storm type effects on super Clausius–Clapeyron scaling of intense rainstorm properties with air temperature. Hydrol Earth Syst Sci. 2015;19(4):1753–66.

[5] Pachauri RKML, editor. Climate change 2014: Synthesis report. Contribution of working groups I, II and III to the fifth assessment report of the intergovernmental panel on climate change2014.

[6] Karl TR, Knight RW. Secular trends of precipitation amount, frequency, and intensity in the United States. Bulletin of the American Meteorological Society. 1998;79(2):231–41.

[7] Karl TR, Knight RW, Plummer N. Trends in high-frequency climate variability in the 20th-century. Nature. 1995;377(6546):217–20.

[8] Higgins RW, Kousky VE. Changes in observed daily precipitation over the United States between 1950–79 and 1980–2009. Journal of Hydrometeorology. 2013;14(1):105–21.

[9] Yan H, Liang C, Li Z, Liu Z, Miao B, He C, et al Impact of precipitation patterns on biomass and species richness of annuals in a dry steppe. PloS One. 2015;10(4):e01253000. 10.1371/journal.pone.0125300 PMC4407894 25906187

[10] Nippert JB, Knapp AK, Briggs JM. Intra-annual rainfall variability and grassland productivity: can the past predict the future? Plant Ecol. 2006;184(1):65–74.

[11] Reyer CPO, Leuzinger S, Rammig A, Wolf A, Bartholomeus RP, Bonfante A, et al A plant's perspective of extremes: terrestrial plant responses to changing climatic variability. Global Change Biol. 2013;19(1):75–89. 10.1111/gcb.12023 PMC3857548 23504722

[12] Grace J, José JS, Meir P, Miranda HS, Montes RA. Productivity and carbon fluxes of tropical savannas. J Biogeogr. 2006;33(3):387–400.

[13] Knapp AK, Briggs JM, Collins SL, Archer SR, Bret-Harte MS, Ewers BE, et al Shrub encroachment in North American grasslands: shifts in growth form dominance rapidly alters control of ecosystem carbon inputs. Global Change Biol. 2008;14(3):615–23.

[14] Tietjen B. Same rainfall amount different vegetation—How environmental conditions and their interactions influence savanna dynamics. Ecol Model. 2016;326:13–22.

[15] Lauenroth WK, Bradford JB. Ecohydrology of dry regions of the United States: precipitation pulses and intraseasonal drought. Ecohydrology. 2009;2(2):173–81.

[16] Knapp AK, Beier C, Briske DD, Classen AT, Luo Y, Reichstein M, et al Consequences of more extreme precipitation regimes for terrestrial ecosystems. Bioscience. 2008;58(9):811–21.

[17] Scholes RJ, Archer SR. Tree-grass interations in savannas Annu Rev Ecol, Evol Syst. 1997;28:517–44.

[18] Wigley BJ, Bond WJ, Hoffman MT. Thicket expansion in a South African savanna under divergent land use: local vs. global drivers? Global Change Biol. 2010;16(3):964–76.

[19] Lett MS, Knapp AK. Woody plant encroachment and removal in mesic grassland: Production and composition responses of herbaceous vegetation. Am Midl Nat. 2005;153(2):217–31.

[20] Buitenwerf R, Bond WJ, Stevens N, Trollope WSW. Increased tree densities in South African savannas: >50 years of data suggests CO2 as a driver. Global Change Biol. 2012;18(2):675–84.

[21] Zhang D-H, Li X-R, Zhang F, Zhang Z-S, Chen Y-L. Effects of rainfall intensity and intermittency on woody vegetation cover and deep soil moisture in dryland ecosystems. Journal of Hydrology. 2016;543, Part B:270–82.

[22] Huxman TE, Wilcox BP, Scott R, Snyder K, Breshears DD, Small E, et al Ecohydrological implications of woody plant encroachment. Ecology. 2005;86:308–19.

[23] Staver AC, Archibald S, Levin S. Tree cover in sub-Saharan Africa: Rainfall and fire constrain forest and savanna as alternative stable states. Ecology. 2011;92(5):1063–72. 21661567 10.1890/10-1684.1

[24] Thomas BF, Behrangi A, Famiglietti JS. Precipitation intensity effects on groundwater recharge in the southwestern United States. Water. 2016;8(3):90.

[25] Smith NG, Schuster MJ, Dukes JS. Rainfall variability and nitrogen addition synergistically reduce plant diversity in a restored tallgrass prairie. J Appl Ecol. 2016;53(2):579–86.

[26] Huang J, Wu P, Zhao X. Effects of rainfall intensity, underlying surface and slope gradient on soil infiltration under simulated rainfall experiments. Catena. 2013;104:93–102.

[27] Van Peer L, Nijs I, Reheul D, De Cauwer B. Species richness and susceptibility to heat and drought extremes in synthesized grassland ecosystems: compositional vs physiological effects. Funct Ecol. 2004;18(6):769–78.

[28] Petrie MD, Collins SL, Litvak ME. The ecological role of small rainfall events in a desert grassland. Ecohydrology. 2015;8(8):1614–22.

[29] Jentsch A, Kreyling J, Beierkuhnlein C. A new generation of climate change experiments: events, not trends. Front Ecol Environ. 2007;5:365–74.

[30] Smith NG, Rodgers VL, Brzostek ER, Kulmatiski A, Avolio ML, Hoover DL, et al Toward a better integration of biological data from precipitation manipulation experiments into Earth system models. Rev Geophys. 2014;52(3):412–34.

[31] Hoover DL, Knapp AK, Smith MD. Resistance and resilience of a grassland ecosystem to climate extremes. Ecology. 2014;95(9):2646–56.

[32] Kulmatiski A, Beard KH. Woody plant encroachment facilitated by increased precipitation intensity. Nature Clim Change. 2013;3(9):833–7.

[33] February EC, Higgins SI, Bond WJ, Swemmer L. Influence of competition and rainfall manipulation on the growth responses of savanna trees and grasses. Ecology. 2013;94:1155–64. 23858655 10.1890/12-0540.1

[34] Du Toit J, Rogers K, Biggs H, editors. The Kruger Experience: Ecology and Management of Savanna Heterogeneity. Washington: Island Press; 2004.

[35] Fisher JB, Whittaker RJ, Malhi Y. ET come home: potential evapotranspiration in geographical ecology. Global Ecol Biogeogr. 2011;20(1):1–18.

[36] Hewitson BC, Crane RG. Consensus between GCM climate change projections with empirical downscaling: precipitation downscaling over South Africa. Int J of Climatol. 2006;26(10):1315–37.

[37] Laprise R, Hernández-Díaz L, Tete K, Sushama L, Šeparović L, Martynov A, et al Climate projections over CORDEX Africa domain using the fifth-generation Canadian Regional Climate Model (CRCM5). Climate Dynamics. 2013;41(11–12):3219–46.

[38] Buitenwerf R, Kulmatiski A, Higgins SI. Soil water retention curves for the major soil types of the Kruger National Park. Koedoe. 2014;56(1).

[39] Mazzacavallo MG, Kulmatiski A. Modelling water uptake provides a new perspective on grass and tree coexistence. PLoS ONE. 2015;10(12):e0144300 doi: 10.1371/journal.pone.0144300 26633177 10.1371/journal.pone.0144300 PMC4669088

[40] Zambatis N, Zacharias PJK, Morris CD, Derry JF. Re-evaluation of the disc pasture meter calibration for the Kruger National Park. Afr J Range For Sci. 2006;23:85–97.

[41] Zeng G, Birchfield ST, Wells CE. Automatic discrimination of fine roots in minirhizotron images. New Phytol. 2008;177(2):549–57. doi: 10.1111/j.1469-8137.2007.02271.x 18042202 10.1111/j.1469-8137.2007.02271.x

[42] Kulmatiski A, Beard KH. Root niche partitioning among grasses, saplings, and trees measured using a tracer technique. Oecologia. 2013;171(1):25–37. doi: 10.1007/s00442-012-2390-0 22752210 10.1007/s00442-012-2390-0

[43] Kulmatiski A, Beard KH, Verweij RJT, February EC. A depth-controlled tracer technique measures vertical, horizontal and temporal patterns of water use by trees and grasses in a subtropical savanna. New Phytol. 2010;188(1):199–209. doi: 10.1111/j.1469-8137.2010.03338.x 20561202 10.1111/j.1469-8137.2010.03338.x

[44] Vendramini PF, Sternberg L. A faster plant stem-water extraction method. Rapid Commun Mass Sp. 2007;21(2):164–8. 10.1002/rcm.2826 17154343

[45] Emerman SH. The tipping bucket equations as a model for macropore flow. Journal of Hydrology. 1995;171(1):23–47.

[46] Tsiko CT, Makurira H, Gerrits AMJ, Savenije HHG. Measuring forest floor and canopy interception in a savannah ecosystem. Physics and Chemistry of the Earth, Parts A/B/C. 2012;47–48:122–7.

[47] Gat JR. Oxygen and hydrogen isotopes in the hydrologic cycle. Annu Rev Earth Planet Sci Lett. 1996;24:225–62.

[48] Warren CP, Kulmatiski A, Beard KH. A combined tracer/evapotranspiration model approach estimates plant water uptake in native and non-native shrub-steppe communities. J Arid Environ. 2015;121(0):67–78.

[49] Schenk HJ. The shallowest possible water extraction profile: A null model for global root distributions. Vadose Zone Journal. 2008;7(3):1119–24.

[50] February EC, Higgins SI. The distribution of tree and grass roots in savannas in relation to soil nitrogen and water. S Afr J Bot. 2010;76(3):517–23.

[51] Wilcox BP. Transformative ecosystem change and ecohydrology: ushering in a new era for watershed management. Ecohydrology. 2010;3(1):126–30.

