# Supplementary material for: A savanna response to precipitation intensity
Source: PLoS One. 2017 Apr 7;12(4):e0175402. doi: 10.1371/journal.pone.0175402 (PMC5384789; doi:10.1371/journal.pone.0175402)
Supplement: S1 Table — (DOCX) [file pone.0175402.s001.docx]

S1 Table. Soil bulk density (g cm^-3^) and soil porosity (cm^3^ cm^-3^) assumed for soils in the experiment.

| Depth | Bulk Density | Porosity |
| --- | --- | --- |
| 10 | 0.8 | 0.70 |
| 20 | 0.9 | 0.66 |
| 30 | 1.0 | 0.62 |
| 40 | 1.1 | 0.58 |
| 75 | 1.2 | 0.55 |
| 100 | 1.3 | 0.51 |
