# Supplementary material for: A savanna response to precipitation intensity
Source: PLoS One. 2017 Apr 7;12(4):e0175402. doi: 10.1371/journal.pone.0175402 (PMC5384789; doi:10.1371/journal.pone.0175402)
Supplement: S1 Fig — Treatment values are modeled assuming that storage tanks dispensed collected ambient precipitation when full. The dotted horizontal line indicates 10 mm events. (DOCX) [file pone.0175402.s002.docx]

S1 Fig. Daily precipitation (mm) in control and treated plots. Treatment values are modeled assuming that storage tanks dispensed collected ambient precipitation when full. The dotted horizontal line indicates 10 mm events.
