# Supplementary material for: A savanna response to precipitation intensity
Source: PLoS One. 2017 Apr 7;12(4):e0175402. doi: 10.1371/journal.pone.0175402 (PMC5384789; doi:10.1371/journal.pone.0175402)
Supplement: S2 Fig — Mean annual plant available soil water (PAW) by depth in one treated and one control plot for the (a) pretreatment and (c-f) subsequent five treatment years. Water was assumed plant available when Ψ > -3 MPa. Values represent the mean PAW across each growing season (October through March). Broadly, treatments, which increased precipitation intensity but not amount, increased deep soil water but did not increase surface (i.e., 5 cm) soil water. (DOCX) [file pone.0175402.s003.docx]

S2 Fig*.* Mean annual plant available soil water (PAW) by depth in one treated and one control plot for the (a) pretreatment and (c-f) subsequent five treatment years. Water was assumed plant available when Ψ > -3 MPa. Values represent the mean PAW across each growing season (October through March). Broadly, treatments, which increased precipitation intensity but not amount, increased deep soil water but did not increase surface (*i.e*., 5 cm) soil water.
