# Supplementary material for: A savanna response to precipitation intensity
Source: PLoS One. 2017 Apr 7;12(4):e0175402. doi: 10.1371/journal.pone.0175402 (PMC5384789; doi:10.1371/journal.pone.0175402)
Supplement: S3 Fig — Comparison of isotope samples measured for (a) Deuterium concentration and (b) Deuterium excess above 18O values and ambient isotope concentrations. Samples were measured repeatedly 3 years apart. (DOCX) [file pone.0175402.s004.docx]

S3 Fig*.* Comparison of isotope samples measured for (a) Deuterium concentration and (b) Deuterium excess above ^18^O values and ambient isotope concentrations. Samples were measured repeatedly 3 years apart.
