# Supplementary material for: A savanna response to precipitation intensity
Source: PLoS One. 2017 Apr 7;12(4):e0175402. doi: 10.1371/journal.pone.0175402 (PMC5384789; doi:10.1371/journal.pone.0175402)
Supplement: S1 File — (DOCX) [file pone.0175402.s005.docx]

**Bd and porosity**

Column A: Depth (10 through 100) in cm

Column B: Bulk density in g cm^-3^

Column C: Porosity (proportion of soil volume) calculated from bulk density assuming a mineral density of 2.65 g cm^-3^

**Daily rainfall**

Column A: Date (month/day/year)

Column B: Ppt (mm rain in control plots)

Column C: Treated ppt (mm rain in treated plots)

Column D: Control (> 2mm precipitation events in control plots)

Column E: Treated (> 2mm precipitation events in treated plots)

**dpm**

Column A: Date (month_day_year)

Column B: Treatment (treated or control)

Columns C-H: The headers (a-f) indicate replicate plot pair name. Values are the height of the disc pasture meter above the ground in cm.

**Dendrometer**

Column A: Plot (1-12) odd plots are treated even plots are control

Column B: Treatment (treated or control)

Column C: Treenum (1-59)

Column D: Remaining column headings are sample dates.  Values are circumference increment between sampling dates in mm.

**Tracers**

Column A: Sample ID (1-2500)

Column B: Date (sample date  Dec or April)

Column C: Depth (depth of injection in cm)

Column D: Treatment (treated or control)

Column E: Plant type (woody or grass)

Column F: Species (species name)

Column G: Delta 18O (ratio of 16 to 18O in delta notation)

Column H: 18 precision (variance in delta 18O among subsamples)

Column I: Delta D (ration of 2H to 1H in delta notation)

Column J: D precision variance in delta D among subsamples)

Column K: D excess (deuterium excess relative to 18O)

Column L: D above control (D excess minus D excess in control samples)

Column M: Proportion uptake (proportion of D excess in the sample relative to other injection depths)

**Rhizotron**

Column A: Year of observation (2010, 2011, 2012 or 2013)

Column B: Trt (control or treated)

Column C: Depth. Depth of observation (cm)

Column D: Number. Number of roots observed five 12.5 by 18 mm windows.

Column E: Number SD. Standard deviation of the number of roots associated with the number of sampling dates in a sampling year.

Column F: Number SE. Standard error of the number of roots associated with the number of sampling dates in a sampling year.

Column G: Area. Mean root area (mm2) in five subsequent 12.5 by 18 mm observation windows.

Column H: Area SD. Standard deviation of the root area associated with the number of sampling dates in a sampling year.

Column I: Area SE. Standard error of the root area associated with the number of sampling dates in a sampling year.
